# Cleft Palate

**Published:** 1872-04

**Authors:** 


### ARTICLE VII.

### *Cleft Palate.*

In this case, admitted into King's College Hospital, there was a fissure of the whole of the soft and two-thirds of the hard palate in a young person. Sir W. Fergusson performed the operation upon the soft palate in the manner which he himself first proposed, dividing the muscles of the soft palate previous to paring the edges of the cleft. Chloroform was administered, and a new form of gag used, which consisted of two grooved plates to fit the teeth of the upper and lower jaws, connected by a horse-shoe-shaped spring; this being placed on the teeth of one side of the mouth, was quite out of the way of the operator during his manipulations. Four sutures were employed to bring the edges of the soft palate accurately into apposition. The sutures were passed in the ordinary way; but an excellent plan is adopted by Sir W. Fergusson, who, to facilitate the adjustment of the sutures, used them of two different colors, passing sutures of the same color on the same side of the cleft, so that one color indicates those to be withdrawn and the other those to be retained. In his remarks after the operation, he referred to the use of chloroform in these operations, and said that the danger of giving much was owing to the loss of sensitiveness of the upper part of the larynx, and the consequent trickling of blood down the trachea and bronchi without corresponding reflex attempts to prevent it. The fact that even after the administration of chloroform some irritation was produced in the larynx and about the palate by the blood, was the cause of the restlessness shown by the patient, but this diminished during the later stage of the operation, when the parts became more tolerant of the cause of excitement in them.—*Medical Times and Gazette.*

---

